# Generation of an isogenic human induced pluripotent stem cell line with a mutant propionyl-CoA carboxylase α subunit

**DOI:** 10.1186/s13023-026-04197-6

**Published:** 2026-01-23

**Authors:** Tianqi Tao, Liwen Lin, Yanyan Tang, Zhenyao Liu, Yu Liu, Yongfang Xie, Xiaohang Hu, Jianli Wang, Tonghe Wang, Guo-Fang Zhang, You Wang, Suhong Zhu

**Affiliations:** 1 Department of Geriatrics, the Second Medical Center and National Clinical Research Center for Geriatric Diseases, Chinese PLA General Hospital, Beijing, China; 2 School of Basic Medicine, Jining Medical University, Jining, Shandong 272067 China; 3 School of Clinical Medicine, Jining Medical University, Jining, Shandong 272067 China; 4 Department of Medical Laboratory, Affiliated Hospital of Jining Medical University, Shandong Key Laboratory of Multi-disciplinary Molecular Diagnosis Precision Medicine, Jining, Shandong China; 5 College of Rehabilitation Medicine, Jining Medical University, Jining, Shandong 272067 China; 6 Sarah W. Stedman Nutrition and Metabolism Center & Duke Molecular Physiology Institute, Duke University, Durham, NC 27701 USA; 7 Department of Medicine, Division of Endocrinology, Metabolism and Nutrition, Duke University Medical Center, Durham, NC 27701 USA; 8 Jining Key Laboratory of Pharmacology, Jining Medical University, Jining, Shandong 272067 China

**Keywords:** Propionic acidemia (PA), Propionyl-CoA carboxylase (PCC), Induced pluripotent stem cells (iPSCs), CRISPR/cas9

## Abstract

**Background:**

Propionic acidemia (PA) is a rare autosomal recessive metabolic disorder caused by defects in propionyl-CoA carboxylase (PCC), a mitochondrial enzyme composed of six alpha (PCCA) and six beta (PCCB) subunits. Mutations in *PCCA/PCCB* genes disrupt PCC function, leading to toxic metabolite accumulation and clinical manifestations. Current research is limited by inadequate patient-derived cellular models and ethical constraints in sample acquisition.

**Method:**

Using CRISPR/Cas9-mediated gene editing, we established an isogenic human induced pluripotent stem cell (iPSC) line carrying the *PCCA* c.2002G> A mutation. The mutant iPSCs were further subjected to directed cardiac differentiation. Characteristic metabolites in the iPSC-derived cardiomyocytes (iPSC-CMs) culture medium were analyzed via untargeted metabolomics, and contractile function was assessed by video-based motion analysis under propionate challenge.

**Results:**

The mutant iPSCs showed sustained expression of pluripotency markers (OCT4, NANOG, SOX-2), maintained normal karyotype (46, XY), and retained trilineage differentiation capacity. Functional characterization demonstrated significantly reduced PCC enzyme activity, accurately modeling PA metabolic pathology. Furthermore, the mutant iPSCs successfully differentiated into cardiomyocytes and exhibited a PA-specific metabolic profile, including significantly elevated propionylcarnitine levels. Upon propionate treatment (2.5 mM), the contractile function of mutant iPSC-CMs was significantly impaired, whereas wild-type iPSC-CMs showed the opposite response with enhanced contraction.

**Conclusions:**

This isogenic iPSC line provides an ethically unconstrained platform to investigate PA molecular mechanisms and genotype-phenotype relationships. The model enables systematic drug screening and therapeutic development while overcoming patient sample limitations.

**Supplementary Information:**

The online version contains supplementary material available at 10.1186/s13023-026-04197-6.

## Introduction

Propionic acidemia (PA) is a rare autosomal recessive metabolic disorder caused by pathogenic variants in the *PCCA* (NM_000282.4) or *PCCB* (NM_000532.4) genes, which encode the α and β subunits of propionyl-CoA carboxylase (PCC), respectively [1]. PCC is a mitochondrial biotin-dependent enzyme essential for the catabolism of propionic acid, propiogenic amino acids (isoleucine, valine, methionine, and threonine), odd-chain fatty acids, and cholesterol [2]. Deficiencies in PCC activity result in the accumulation of propionyl-CoA and its toxic metabolites (e.g., 3-hydroxypropionate and methylcitrate), leading to severe clinical manifestations including metabolic acidosis, hyperammonemia, cardiomyopathy, and neurodevelopmental impairment [3, 4]. The pathophysiology of PA is driven by the disruption of propionyl-CoA metabolism, particularly the impaired conversion of propionyl-CoA to methylmalonyl-CoA, which causes mitochondrial dysfunction and cellular damage through multiple mechanisms, including inhibition of the tricarboxylic acid cycle and aberrant post-translational modifications [5].

Murine models (e.g., *Pcca*^-/-^ (A138T)) remain the primary PA research tool but retain 10–15% residual PCC activity—far exceeding the < 2% levels seen in severe human cases [6, 7]. This discrepancy fundamentally limits their ability to model critical disease manifestations, such as severe metabolic crises and cardiomyopathy. While patient-derived induced pluripotent stem cells (iPSCs) have emerged as a promising tool for disease modeling, as demonstrated by numerous studies [8–11], their application is limited by challenges such as reliance on donor availability, ethical complexities, and limited genetic diversity. To maximize the clinical relevance of our model for the Chinese population, we targeted the PCCA c.2002G> A (p.Gly668Arg) mutation. This variant represents the most frequently reported pathogenic mutation in Chinese PA patients, accounting for approximately 22% of PCCA-associated cases [4, 12, 13, 14], and is associated with severe clinical manifestations. We hypothesize that by editing the genes of wild-type iPSCs based on known PA gene mutations, we can generate PA-specific stem cell lines. These lines could then be differentiated into various organ types, offering a valuable model for further investigation of PA.

In this study, we aim to utilize CRISPR/Cas9 gene editing technology to introduce the *PCCA* c.2002G> A mutation into normal iPSCs, thereby generating an isogenic iPSC-based disease model for PA. These genetically edited iPSCs will be differentiated into disease-relevant cell types, such as cardiomyocytes, to investigate metabolic abnormalities and contractile dysfunction associated with PA, offering a robust platform to investigate the underlying molecular mechanisms of PA and to explore potential therapeutic interventions. This approach not only addresses the limitations of existing models but also provides a patient-specific tool for advancing PA research and treatment development.

## Materials and methods

### Cell culture

The iPSCs (DYR0100, Cell Bank/Stem Cell Bank, Chinese Academy of Sciences) were cultured on a 6-well plates coated with Matrigel (354277, Corning) in ncTarget medium (RP01020, Shownin Biotechnology Co., Ltd., China) in a humidified incubator at 37 °C with 5% CO_2_. Cells were passaged at a ratio of 1:12 upon reaching approximately 80% confluence using Gentle Cell Dissociation Reagent (100–0485, STEMCELL). Prior to proceeding with subsequent experiments, cell lines were confirmed to be free of bacterial and mycoplasma contamination.

### CRISPR Cas9-mediated PCCA mutation and Sanger sequencing

Mutation of PCCA in iPSCs was accomplished by Cyagen (China). Briefly, Cas9 protein and gRNA(gRNA-A1: CCACCACCACTCCGGGCATC-GGG) were incubated to assemble the RNP complex, which was subsequently introduced into cells via electroporation along with the Oligo (Smart-CRISP™ cell gene editing system, Cyagen, China). Following electroporation, a cell pool was selected based on the HDR efficiency for monoclonal preparation. The selected monoclonal cell lines were then validated using PCR. Eventually, the Sanger sequencing was employed for homozygote verification.

### Off-target analysis

To evaluate the potential off-target effects of CRISPR/Cas9 genome editing, the sgRNA sequence (A1: CCACCACCACTCCGGGCATC-GGG) was analyzed using the CCTop prediction tool. The top 10 predicted off-target loci were selected based on sequence similarity and off-target scoring. Specific primers were designed for each site (listed in Supplementary Table S1).

Genomic DNA was extracted from both the wild-type iPSCs and the CRISPR/Cas9-edited iPSC clone carrying the homozygous PCCA c.2002G > A mutation (designated as clone 1H10, hereafter referred to as mutant iPSCs). Each target locus was amplified by PCR and Sanger sequencing was performed. Chromatograms were aligned using SnapGene software and manually inspected for any mutations, including base substitutions, insertions, or deletions.

### Cell viability assay

Cells were seeded at a density of 5 × 10^3^ cells per well in 96-well plates. Following the designated treatments, the cells were incubated with CCK-8 reagent (CT0001, Sparkjade Biotechnology Co., Ltd., China) in a 5% CO_2_ incubator at 37 °C for 1 hour. The absorbance at 490 nm was then measured using the SpectraMax Plus 384 Microplate Reader (PLUS 384, Molecular Devices, USA). Absorbance values is proportional to the cell viability.

### Western blot assay

The protein concentration of the cellular lysates was quantified using the BCA Protein Assay kit (GK10009, GLPBIO). Fractions were resolved on 4%-20% SDS-PAGE precast gels (P0468S, Beyotime) with 40 µg of protein per lane, and subsequently transferred onto PVDF membranes. The membranes were blocked with 5% BSA in Tris-bufered saline containing 0.1% Tween-20 (TBS-T) at 4 °C for 4 h. The membranes were then probed with primary antibodies, including anti-PCCA (1:1,000 dilution, 21988–1-AP, proteintech), anti-calreticulin (1:1,000 dilution, ab92516, Abcam), anti-propionyllysine (1:500 dilution,PTM-201, PTM Biolabs), and anti-GAPDH (1:2000 dilution, ab9485, Abcam) at 4 °C overnight. Goat anti-Rabbit IgG (H+L)-HRP (1:4000, AS014, ABclonal) were used as the secondary antibodies at a dilution of 1:4000 for blotting for 1 hour at room temperature. Protein bands were visualized using an enhanced chemiluminescence detection system. Band optical densities were quantified using ImageJ software. The densitometry results for PCCA and Calreticulin (CRT) were normalized to GAPDH, while pan-propionyllysine levels were normalized to the Coomassie Blue-stained gel.

### Real-time PCR assay

Real-time polymerase chain reaction (RT-PCR) was performed using an LC480 platform (Roche Applied Science, Mannheim, Germany). SYBR Green qPCR Mix (D7260, Beyotime) was used to detect and quantify the expression level of the target gene. GAPDH served as an internal control. The following primers were used:

PCCA:

5′-GCAAGAAGATGGGCATTAAGACA-3′ (F),

5′-GCCAACACAGACAGCCTCAT-3′ (R);

GAPDH:

5′-GGAGCGAGATCCCTCCAAAAT-3′ (F),

5′-GGCTGTTGTCATACTTCTCATGG-3′ (R).

F, forward; R, reverse.

### Immunocytochemistry for pluripotency and differentiation markers

iPSCs were seeded in 24-well plates coated with Matrige. When reaching above 80% confluence, cells were fixed with 4% frozen paraformaldehyde (PFA) for 30 min, then washed three times with PBS three times with PBS (2 minutes each) and made permeable using 0.4% Triton X-100 in PBS for 20 minutes, then cells were treated with 5% BSA in TBST for 60 minutes at room temperature.

This was followed by overnight incubation at 4 °C with primary antibodies targeting pluripotency markers: anti-Nanog (1:1000, ab109250, Abcam), anti-Oct4 (1:1000, ab200834, Abcam), and anti-SOX2 (1:1000, ab93689, Abcam); as well as differentiation markers: anti-PAX6 (1:350, ab195045, Abcam), anti-Brachyury (1:1000, ab209665, Abcam), and anti-SOX17 (1:3000, ab224637, Abcam). After primary antibody incubation, cells were washed with PBS three times (5 minutes each) and incubated with Goat anti-Rabbit IgG H&L (Alexa Fluor^®^ 594 or Alexa Fluor^®^ 488, Abcam) for 60 minutes at room temperature. Cells were then washed three additional times with PBS (10 minutes each), and nuclei were counterstained with 4’,6-diamidino-2-phenylindole (DAPI). Fluorescent images were acquired immediately using a Nikon Ti2 fluorescence microscope or the C×7 High-Content Analysis System (Thermo Fisher Scientific, USA).

### Karyotype analysis

Karyotyping analysis was performed to assess the chromosomal integrity of iPSCs. Cells were cultured to reach an 80% confluence, then treated with colchicine (0.3 ng/mL) to arrest them in metaphase. After harvesting, cells were hypotonicized using 0.075 M KCl to promote cell swelling, followed by fixation in methanol acid (3:1). Fixed cells were dropped onto glass slides and stained using Giemsa solution for chromosome visualization. The karyotypes were automatically captured by the automatic scanning system GSL120 (Leica Biosystems Richmond, Inc., USA). Chromosomal karyotypings were described according to the International System for Human Cytogenetic Nomenclature (ISCN, 2020).

### Embryoid body (EB) formation

iPSCs were cultured to above 80% confluence. Cells were dissociated into single-cell suspensions using Solase (RP01021, Shownin Biotechnology Co., Ltd., China) at 37 °C for 6–8 minutes, followed by cell counting. The single-cell suspension was then seeded at a density of 80,000– 120,000 cells/cm^2^ in T25 culture flasks. The cells were incubated at 37 °C on a Variable Speed Tube Revolver (Crystal, USA) with 10 rpm for 24 hours to facilitate embryoid body (EB) formation, images were acquired immediately using a Nikon Ti2 fluorescence microscope.

### iPSCs PCC activity assay

The protocol for measuring PCC activity was adapted from previously established method [11, 15]. Approximately 10^7^cell were harvested for homogenized in 1 ml of a 50 mM potassium phosphate buffer (pH 7.4). Around 40 µg cellular protein was used for the assay, to which 100 μl of an enzyme reaction mixture was added. This mixture consisted of 100 mM Tris-HCl (pH 7.5), 5 mM MgCl_2_, 1 mM DTT, 10 mM KCl, 40 mM NaHCO_3_, 1 mM Biotin, and 6 mM ATP. The reaction was initiated by adding 10 μl of 11.8 mM propionyl-CoA (P5397, sigma) and carried out at 37 °C for 40 min. After incubation, 10 μl of the reaction mixture was combined with 10 μl of 0.01 mM M2 Acetyl-CoA (658650, sigma) as an internal standard (IS), followed by mixing with 50 μl of 200 mM formic acid to terminate the reaction. The mixture was then centrifuged at 14,000 × rpm for 10 min. The supernatants were analyzed using LC-5500 QTRAP-MS/MS (AB SCIEX). Ion chromatograms of methylmalonyl-CoA, and M2 Acetyl-CoA were extracted and quantified based on m/z values of 868/361 and 812/305, respectively.

### Cardiac differentiation of iPSCs

When the cells reached approximately 80% confluence, cardiac differentiation was induced using a commercially available cardiomyocyte differentiation kit (Cellapy, CA2004500, Beijing, China) according to the manufacturer’s instructions. Spontaneous beating was observed from day 8 post-induction. By day 12, a synchronously beating monolayer syncytium of iPSC-CMs had formed in a networked structure. Subsequently, iPSC-CMs were purified using purification medium (Cellapy, CA2005100, Beijing, China).

### Metabolomic profiling of iPSC-cardiomyocyte medium

Metabolite profiling of the cell culture medium was conducted using ultra-high performance liquid chromatography coupled with tandem mass spectrometry (UPLC-Q-Exactive Plus MS, Thermo Scientific) in a non-targeted metabolomics approach. Cardiomyocyte-like cells differentiated for 12 days were purified over a 2-day period, after which the medium was replaced with fresh cardiomyocyte maintenance medium (Cellapy, CA2015500, Beijing, China) and cultured for an additional 48 hours. Subsequently, 2 mL of medium was collected and centrifuged at 1000 × g for 3 min at 4 °C, rapidly frozen in liquid nitrogen for 1 min, and stored at −80 °C until further analysis.

For metabolite extraction, samples were thawed at 4 °C and vortexed. A 1 mL aliquot was transferred to a microcentrifuge tube and dried under vacuum centrifugation. The residue was reconstituted in 1 mL of ice-cold methanol-water (4:1, v/v), vortex-mixed, and sonicated in an ice bath for 20 min. After incubation at −20 °C for 1 h, the samples were centrifuged at 16,000 × g for 20 min at 4 °C. The supernatant was collected and dried again. For LC–MS analysis, the dried extract was reconstituted in 100 μL of ice-cold methanol-water (1:1, v/v), centrifuged at 20,000 × g for 15 min at 4 °C, and the resulting supernatant was injected into the system.

Chromatographic separation was performed on an ACQUITY UPLC® HSS T3 column (2.1 × 100 mm, 1.8 μm; Waters, Milford, MA, USA) using a gradient elution program with mobile phase A (0.1% formic acid in water) and B (0.1% formic acid in acetonitrile). Mass spectrometry was performed using a Q-Exactive Plus Orbitrap mass spectrometer (Thermo Scientific) equipped with a HESI ion source in both positive and negative electrospray ionization (ESI) modes. The ion source parameters were set as follows: spray voltage, 3.8 kV (positive) and 3.2 kV (negative); capillary temperature, 320 °C; sheath gas flow rate, 30 arbitrary units; auxiliary gas flow rate, 5 arbitrary units; probe heater temperature, 350 °C; and S-Lens RF level, 50. Full MS scans were acquired across the m/z range of 75–1050, and data-dependent acquisition (DDA) was employed for MS/MS fragmentation.

Raw data were processed using MS-DIAL software for peak picking, alignment, and metabolite identification by matching against HMDB, MassBank, and other public databases. Peaks with more than 50% missing values in any group were excluded. Data from both ionization modes were normalized to total ion current and subjected to multivariate statistical analysis.

### Assessment of contractile function in iPSC-derived cardiomyocytes

On day 12 of differentiation, when the iPSC-CMs had formed a synchronously beating monolayer syncytium, contractile activity was recorded. Videos were captured under baseline conditions (50 frames per second, 10 seconds duration). The culture was then returned to the incubator for 10 minutes to re-equilibrate, followed by treatment with 2.5 mM propionic acid and another 10-minute incubation. Post-treatment videos were recorded under identical settings. Contractility was analyzed using the MUSCLEMOTION plugin for ImageJ.

### Statistical methods

All data are expressed as the means ± standard deviations (SD). A Student’s *t*-test was used for comparisons between two groups, while a one-way ANOVA test was conducted for comparisons among more than two groups. *p* values < 0.05 were considered to indicate a statistically significant difference. In each experiment, at least three replicates were used for statistical analysis. Statistical differences were analyzed using Prism software.

## Results

### Generation and characterization of iPSCs harboring a homozygous missense mutation in *PCCA*

**Fig. 1:**
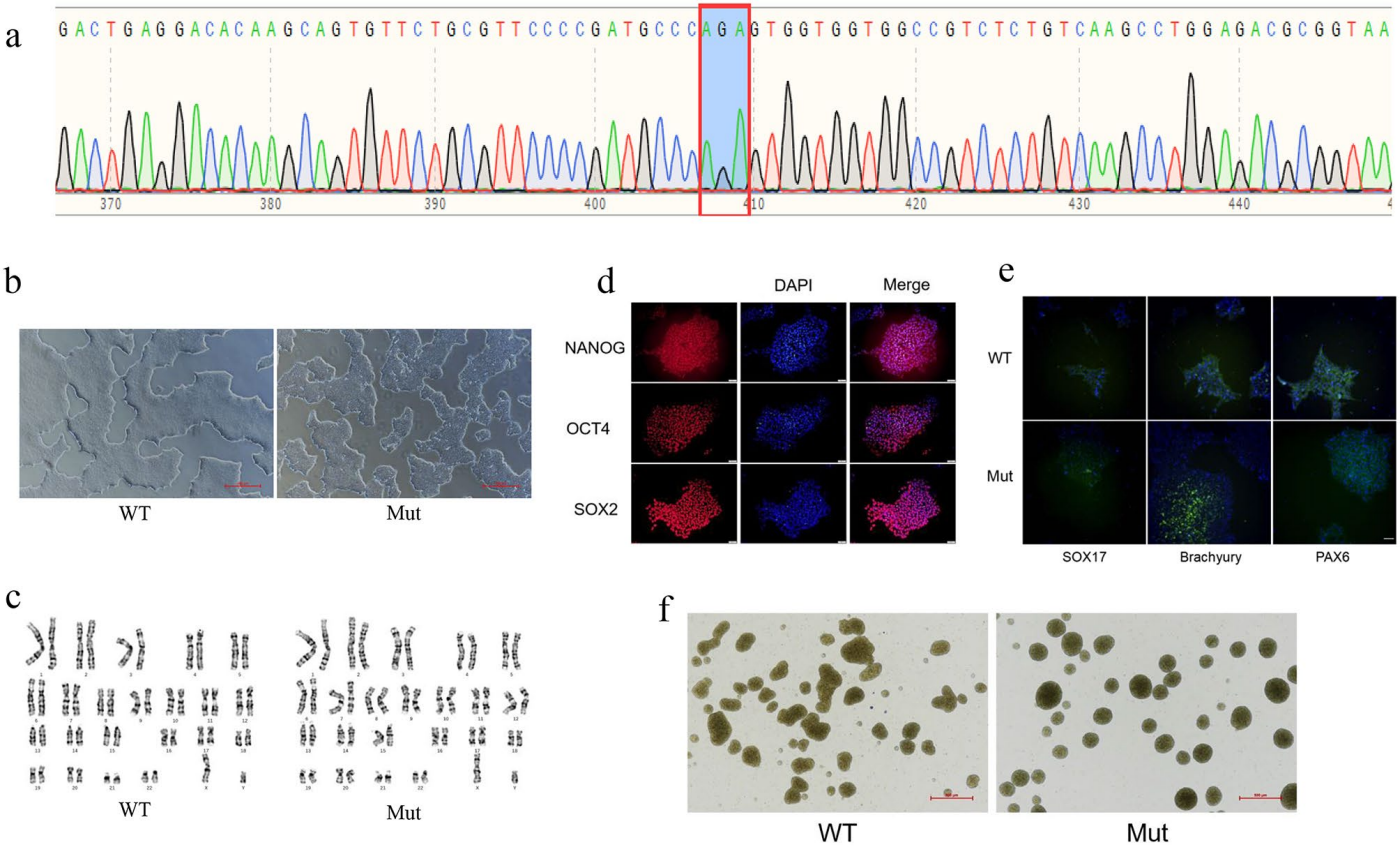
Characterization of *PCCA*-mutant induced pluripotent stem cells (iPSCs). (**a**) Sanger sequencing chromatogram showing the homozygous c0.2002 G > A mutation in the *PCCA* gene. (**b**) microscopy image of iPSC colony morphology (scale bar = 500 μm). (**c**) G-banding karyotype analysis. (**d**) immunofluorescence staining of pluripotency markers OCT4, NANOG, and SOX2 (scale bar = 50 μm). (**e**) immunofluorescence staining of trilineage differentiation markers SOX17, Brachyury, and PAX6 (scale bar = 50 μm). (**f**) Embryoid body formation (scale bar = 500 μm)

In this study, we generated induced pluripotent stem cells (iPSCs) harboring a specific mutation in the *PCCA* gene using CRISPR/Cas9-mediated gene editing. The targeted mutation, c.2002G > A (p.Gly668Arg), was introduced into exon 22 of the *PCCA* gene, resulting in a glycine-to-arginine substitution at position 668 (GGA to AGA). Sanger sequencing confirmed the successful introduction of the desired mutation in the iPSCs, with no detectable off-target effects (Fig. 1a).

Off-target analysis by PCR and Sanger sequencing revealed no sequence alterations at the top 10 predicted off-target loci in the PCCA-mutant iPSCs (clone 1H10) compared with wild-type iPSCs (see Supplementary Figure S1). All chromatograms showed perfect alignment across samples. These results confirm that the CRISPR/Cas9 editing strategy exhibited high specificity and did not induce off-target genomic changes in the tested regions.

Under standard culture conditions, the *PCCA*-mutant (Mut) iPSCs exhibited typical iPSC morphology, forming compact colonies with well-defined edges, and demonstrated growth kinetics and doubling times comparable to those of wild-type (WT) iPSCs (Fig. 1b). G-banding karyotype analysis revealed a normal diploid karyotype (46, XY) in the mutant iPSCs, consistent with the karyotype of WT iPSCs, indicating genomic stability (Fig. 1c).

To assess pluripotency, immunofluorescence staining was performed, confirming robust expression of key pluripotency markers, including octamer-binding transcription factor 4 (OCT4), homeobox protein NAOG (NANOG), and SRY-box transcription factor 2 (SOX2), in the mutant iPSCs (Fig. 1d). Furthermore, the differentiation potential of the mutant iPSCs was evaluated through immunofluorescence staining of trilineage markers: SOX17 (endoderm), Brachyury (mesoderm), and PAX6 (ectoderm). The results demonstrated the ability of the mutant iPSCs to differentiate into cell types representing all three germ layers (Fig. 1e). Additionally, embryoid body (EB) formation assays confirmed the capacity of the mutant iPSCs to undergo spontaneous differentiation (Fig. 1f).

Collectively, these findings indicate that the *PCCA*-mutant iPSCs maintain normal pluripotency, genomic integrity, and differentiation potential, providing a reliable model for further investigation of PA.

### Unaltered PCC expression and endoplasmic reticulum stress in mutant iPSCs

**Fig. 2:**
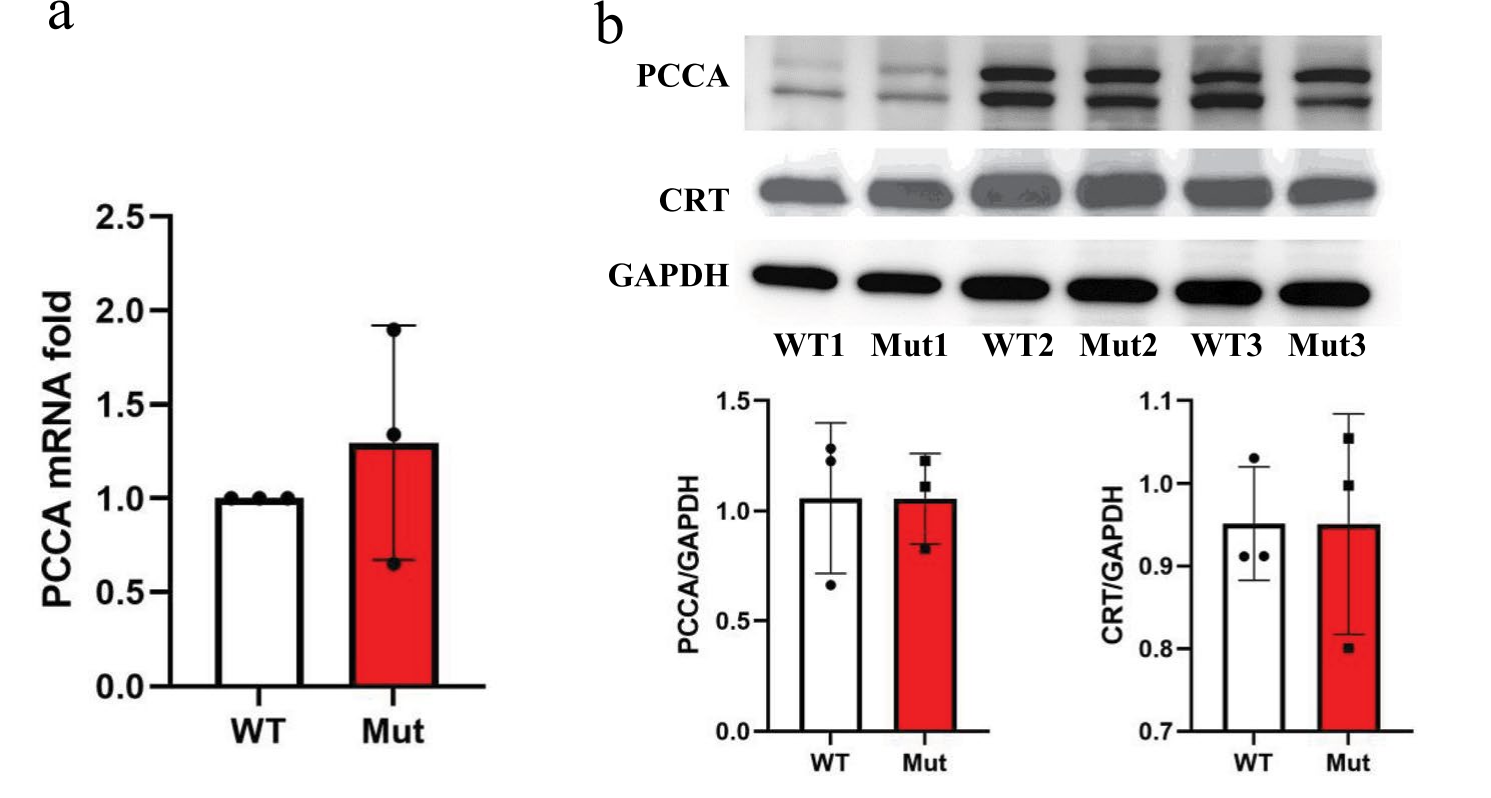
The expression of PCCA and CRT in mutant iPscs. (**a**) quantitative analysis of *PCCA* mRNA expression levels. (**b**) Western blot analysis of PCCA and CRT protein levels in wild-type (WT) and mutant (Mut) iPscs. *N* = 3

To evaluate the molecular consequences of the *PCCA* c.2002G > A mutation, we analyzed the expression of *PCCA* mRNA and protein in mutant iPSCs compared to wild-type (WT) controls. Quantitative reverse transcription polymerase chain reaction (qRT-PCR) revealed no significant differences in PCCA mRNA levels between mutant and WT iPSCs (*p* > 0.05), indicating that the mutation does not disrupt transcriptional regulation of the PCCA gene (Fig. 2 a). Similarly, Western blot analysis demonstrated comparable levels of PCCA protein in both mutant and WT iPSCs, suggesting that the p.Gly668Arg substitution does not impair protein stability or translation efficiency (Fig. 2 b).

Given the potential for gene mutations to alter the primary structure of proteins, leading to misfolded proteins that could induce endoplasmic reticulum (ER) stress, we further investigated the expression of the ER stress marker calreticulin. Western blot analysis showed no significant differences in calreticulin protein levels between mutant and WT iPSCs (*p* > 0.05), indicating that the PCCA mutation does not trigger detectable ER stress under basal culture conditions (Fig. 2 b).

These results demonstrate that the *PCCA* c.2002G > A mutation does not alter *PCCA* transcript or protein expression levels and does not induce ER stress in mutant iPSCs.

### Exogenous propionate induces vulnerability in mutant iPSCs

To evaluate the functional impact of the *PCCA* c.2002G > A mutation under similar metabolic stresses found in PA, wild-type iPSCs (WT) and mutant iPSCs (Mut) were exposed to exogenous propionate, followed by analysis of cellular viability and global protein propionylation.

**Fig. 3:**
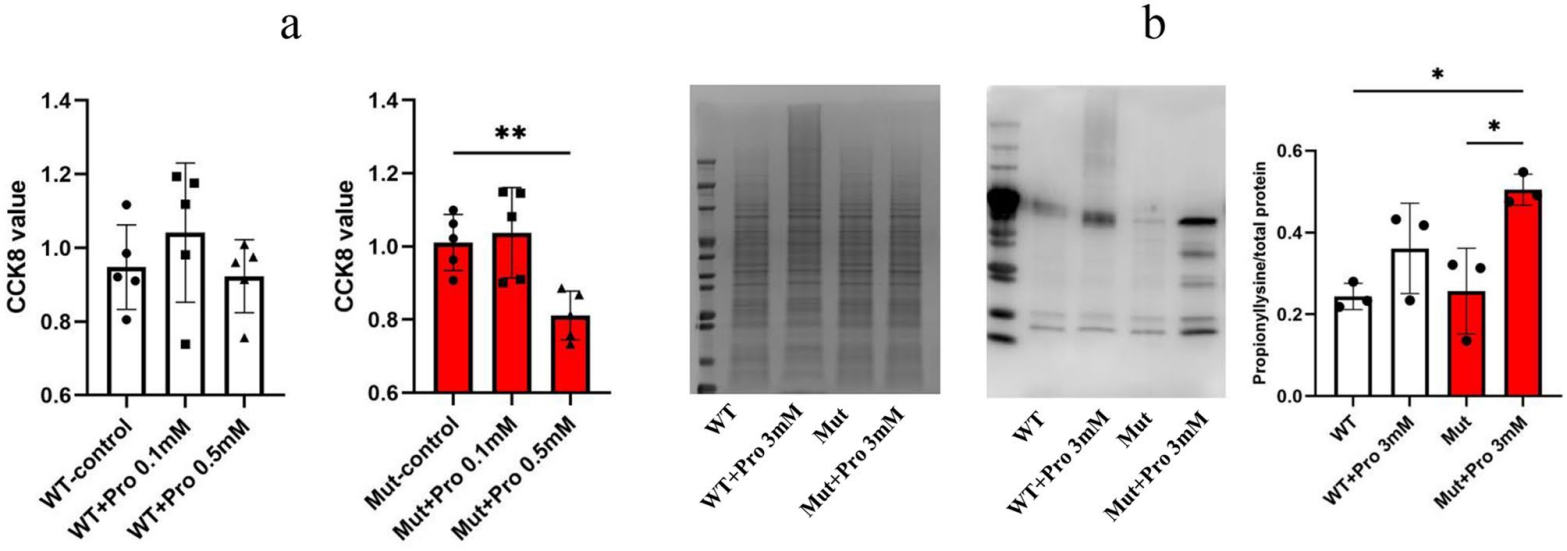
Effects of exogenous propionic acid (pro) treatment on iPSCs. (**a**) the viability of WT and Mut iPscs exposed to varying doses of Pro; (**b**) propionylation profile analysis: left panel shows Coomassie Blue staining of total proteins as loading control; middle panel is a representative image of total protein propionylation levels detected using an anti-propionyllysine antibody. *N* = 3. * and ** denote signifcant diferences between indicated groups with *p* < 0.05 and *p* < 0.01

The mutant iPSCs exhibited pronounced vulnerability to propionate treatment. At a concentration of 0.5 mM, the viability of the mutant iPSCs was significantly reduced compared to the untreated group (Mut-control: 1.01 ± 0.08 vs. Mut-0.5 mM: 0.81 ± 0.07, *p* < 0.01). In contrast, WT iPSCs showed no significant viability reduction at this concentration (WT-0.5 mM: 0.92 ± 0.10 vs. WT-control: 0.95 ± 0.11; *p* > 0.05), demonstrating resistance to propionate-induced cytotoxicity (Fig. 3 a).

Then, we analyzed total cellular protein propionylation using an anti-propionyllysine antibody. Untreated Mut exhibited baseline propionylation levels comparable to WT. However, exposure to 3 mM propionate, no significant difference was observed between treated and untreated WT cells. In contrast, treated mutant iPSCs showed a 1.9-fold increase in total protein propionylation compared to untreated mutant iPSCs (Mut- 3 mM: 0.50 ± 0.04 vs. Mut: 0.26 ± 0.1, *p* < 0.05). This indicates that mutant iPSCs are more susceptible to propionate exposure (Fig. 3 b).

### The *PCCA* c.2002G > A mutation impairs PCC activity

**Fig. 4:**
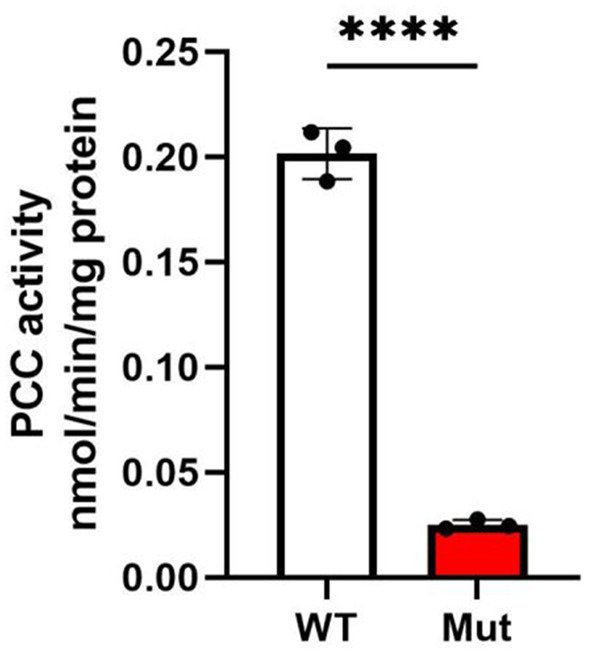
PCC (propionyl-CoA carboxylase) activities in iPscs from wild type and *PCCA* mutant cells. *N* = 3. The error bar represents the SE. **** denote signifcant diferences between indicated groups with *p* < 0.0001

To assess the impact of the genetic mutation on PCC enzyme activity, we performed in vitro enzyme activity assays. Mutant iPSCs exhibited a profound 8-fold reduction in PCC activity compared to WT controls (0.025 ± 0.002 vs. 0.20 ± 0.01, *p* < 0.0001), closely mirroring the < 2% residual activity observed in severe PA patients [3]. These findings suggest a significant impairment of PCC enzyme activity in the mutant iPSCs compared to the WT (Fig. 4).

### Mutant iPSCs successfully differentiate into cardiomyocytes

To validate the cardiogenic potential of the mutant iPSC line, directed cardiac differentiation was performed. By day 12 of differentiation, both wild-type (WT) and mutant (Mut) iPSCs had successfully formed synchronously beating syncytia with a networked structure (Supplementary Videos S1, S2).

### Characteristic metabolites in iPSC-CMs

**Fig. 5:**
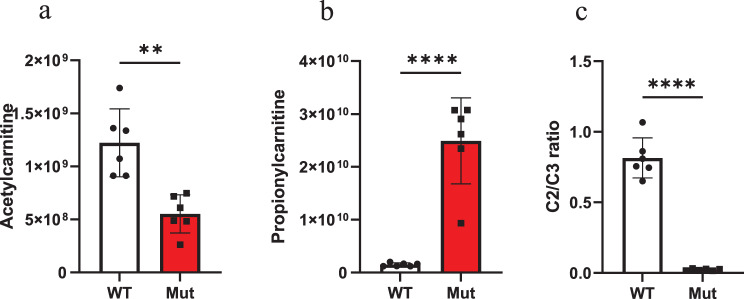
Characteristic metabolites in the culture medium of iPSC-derived cardiomyocytes (iPSC-CMs) from wild type and PCC mutant groups. (**a**) relative abundance of acetylcarnitine. (**b**) relative abundance of propionylcarnitine. (**c**) ratio of acetylcarnitine (C2) to propionylcarnitine (C3) relative abundance. *N* = 6. The error bar represents the SE. ** and **** indicate significant differences between groups with *p* < 0.01 and *p* < 0.0001, respectively

To investigate the impact of the *PCCA* mutation on metabolic activity, we performed targeted analysis of characteristic metabolites in the cell culture medium. Compared with WT iPSC-CMs, the mutant line exhibited a 2.2-fold decrease in acetylcarnitine (Mut: 5.52 × 10^8^ ± 1.80 × 10^8^ vs. WT: 1.22 × 10^9^ ± 3.21 × 10^8^) and a 16.5-fold increase in propionylcarnitine (Mut: 2.49 × 10^1 0^ ± 8.13 × 10^9^ vs. WT: 1.51 × 10^9^ ± 3.31 × 10^8^). The acetylcarnitine-to-propionylcarnitine ratio was markedly reduced by approximately 40-fold (Mut: 0.02 ± 0.0067 vs. WT: 0.81 ± 0.14). These results indicate that the *PCCA* mutation disrupts propionate metabolism, leading to significant accumulation of propionylcarnitine in iPSC-CMs (Fig. 5).

### Propionate impairs contractility in mutant iPSC-CMs

**Fig. 6:**
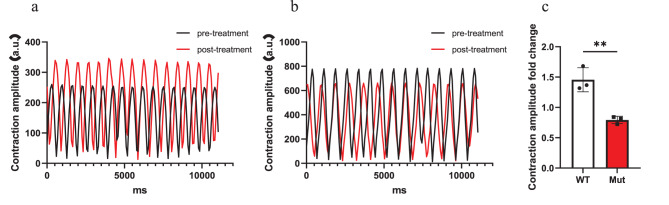
Effect of propionate (PA) on the contraction amplitude of iPSC-derived cardiomyocytes (iPSC-CMs). Contraction amplitude is expressed as the post- to pre-treatment ratio after 10 minutes of exposure to 2.5 mM PA. (**a**) Representative contraction traces of wild-type (WT) iPSC-CMs in response to PA. (**b**) Representative contraction traces of mutant (Mut) iPSC-CMs in response to PA. (**c**) statistical summary of contraction amplitude ratios. *N* = 3. The error bar represents the SE. **indicate significant differences between groups with *p* < 0.01

To assess the effect of propionate on the contractile function of iPSC-CMs, we analyzed cardiomyocyte contraction videos [16]. The contraction amplitude of WT iPSC-CMs increased following propionate treatment, whereas that of Mut iPSC-CMs markedly decreased. The post- to pre-treatment ratio of contraction amplitude was significantly lower in Mut iPSC-CMs than in WT cells (Mut: 0.79 ± 0.06 vs. WT: 1.46 ± 0.20), representing an approximately 1.8-fold reduction in the Mut group compared to WT. These results demonstrate that the PCCA mutation exacerbates the susceptibility of cardiomyocytes to propionate-induced contractile dysfunction (Fig. 6, Supplementary Videos S3, S4).

## Discussion

PA is predominantly caused by mutations in the *PCCA* or *PCCB* genes, exhibits significant genetic heterogeneity across populations. In Chinese PA cohorts, the *PCCA* c.2002G > A (p.Gly668Arg) mutation represents the most frequently reported pathogenic variant, accounting for around 22% of *PCCA*-associated cases [4, 12, 13, 14]. This mutation, located in exon 22, disrupts a highly conserved glycine residue (Gly668) within the biotin carboxylase domain of PCC—a region critical for substrate binding and enzymatic dimerization [5]. Clinically, this variant is associated with severe disease manifestations, including recurrent metabolic decompensation, hypertrophic cardiomyopathy, and progressive neurological impairment [4, 12]. In this study, we established a CRISPR/Cas9-edited *PCCA* c.2002G > A iPSC model to characterize the molecular and metabolic hallmarks of PA.

To ensure the safety and specificity of our gene editing approach, we performed off-target analysis based on CCTop-predicted sgRNA binding sites, followed by PCR and Sanger sequencing validation of the top 10 candidates. No off-target mutations were detected, indicating that our CRISPR/Cas9 strategy maintains high precision within the DYR0100 iPSC genomic background. These results align with prior studies and support the robustness of our isogenic iPSC model for downstream applications such as disease modeling and therapeutic screening.

Our functional characterization revealed a critical outcome: mutant iPSCs exhibited an 8-fold reduction in PCC enzyme activity compared to WT controls (*p <* 0.0001, Fig. 4). This severe enzymatic deficiency aligns with the biochemical phenotype of PA patients and confirms the pathogenicity of the c.2002G > A mutation. Notably, the mutation did not alter *PCCA* mRNA or protein levels (Fig. 2 a-b), nor did it induce endoplasmic reticulum (ER) stress, as evidenced by unchanged calreticulin expression (Fig. 2 b). These findings suggest that the p.Gly668Arg substitution primarily disrupts PCC catalytic function rather than affecting transcriptional regulation, protein expression, or spatial conformation.

Under basal culture conditions, mutant iPSCs displayed normal pluripotency, karyotype, and differentiation capacity (Fig. 1a-f). This phenotypic stability is likely relevant to the low abundance of propiogenic precursors (e.g., odd-chain fatty acids, propiogenic amino acids) in standard iPSC media. However, exposure to exogenous propionate revealed the metabolic defect of mutant iPSCs: at 0.5 mM, viability decreased significantly (*p* < 0.01, Fig. 3 a), while WT cells remained unaffected. This hypersensitivity mirrors the clinical necessity for propiogenic amino acids-free or propiogenic amino acids-reduced formula feeding in children with PA [17–19].

Furthermore, propionate treatment induced a 1.9-fold increase in total protein propionylation in mutant iPSCs (*p*  < 0.05, Fig. 3b), a phenomenon attributed to PCC deficiency and subsequent propionyl-CoA accumulation. Propionylation, a post- -translational modification analogous to acetylation, is catalyzed by the transfer of a propionyl group from propionyl-CoA to lysine residues on target proteins, including histones and metabolic enzymes [20]. The observed increase in propionylation in our mutant iPSCs reflects the impaired metabolism of propionyl-CoA due to the PCCA mutation, leading to its accumulation and subsequent aberrant modification of cellular proteins.

Emerging evidence highlights protein propionylation as a key mediator of cellular dysfunction in PA. In patient-derived fibroblasts and hepatocytes, pathological propionyl-CoA accumulation drives excessive protein propionylation, impairing mitochondrial complex I activity and respiration [21]. Propionylation also disrupts epigenetic regulation. In PA mice, propionate-induced histone H3 lysine propionylation (H3K23pr) synergizes with hyperacetylation to dysregulate cardiac genes like Pde9a and Mme, leading to diastolic dysfunction in females [22]. Our findings align with these reports, as the elevated propionylation levels in mutant iPSCs correlate with the metabolic burden of propionyl-CoA and its detrimental effects on cellular function. The observed increase in protein propionylation not only serves as a biomarker of metabolic stress but also provides mechanistic insights into the cellular toxicity associated with PCC deficiency.

Our study demonstrates that iPSCs carrying the *PCCA* c.2002G > A mutation can successfully differentiate into cardiomyocytes that recapitulate key metabolic features of PA. Upon exogenous propionate challenge, WT cardiomyocytes exhibited enhanced contractility, likely attributable to the utilization of propionate—a short-chain fatty acid—as an energetic substrate supporting cardiac contraction. In contrast, mutant cardiomyocytes displayed pronounced contractile dysfunction, possibly resulting from the inability to metabolize propionate, leading to disrupted fatty acid oxidation and compromised energy production according to our previous work [23, 24]. These findings not only elucidate the pathophysiological link between propionate metabolism and cardiac function in PA but also provide a robust human iPSC-based model for further investigating disease-specific cardiac complications and screening potential therapeutic interventions. While the present study focused on metabolic and functional characterization, future investigations employing transcriptomic profiling, such as RNA sequencing, could provide further insights into the molecular pathways and gene expression alterations underlying the contractile and metabolic dysfunctions observed in PA cardiomyocytes.

## Conclusion

In this study, we successfully developed an isogenic iPSC model harboring the *PCCA* c.2002G > A mutation, which recapitulates the metabolic hallmarks of PA while maintaining pluripotency and differentiation capacity. This model enables a precise dissection of PCC deficiency without confounding genetic variables, as demonstrated by the 8-fold reduction in PCC enzyme activity and preserved transcriptional and translational fidelity. Notably, the iPSC model can be differentiated into disease-relevant cell types, including hepatocytes for metabolic studies, neurons for neurotoxicity assessments, and cardiomyocytes for PA-related cardiomyopathy research and high-throughput drug screening. Specifically, we demonstrated that the mutant iPSCs can robustly differentiate into functional cardiomyocytes that exhibit characteristic PA metabolic profiles, including significantly elevated propionylcarnitine and a reduced acetylcarnitine-to-propionylcarnitine ratio. Furthermore, under exogenous propionate challenge, the mutant cardiomyocytes showed impaired contractility, in contrast to the enhanced contraction observed in WT cells, reflecting metabolic stress from accumulated propionyl-CoA, further exacerbated by external propionate. This provides a unified platform for investigating the tissue-specific pathophysiology of PA.

Our findings reveal that the PCCA mutation impairs PCC activity, leading to increased protein propionylation and cellular vulnerability under propionate-induced metabolic stress. This model offers a robust and versatile platform for further investigation into the molecular mechanisms underlying PA and for exploring potential therapeutic interventions. By enabling the differentiation of iPSCs into disease-relevant cell types, this research contributes valuable insights into the tissue-specific pathophysiology of PA. Moreover, this study highlights the potential of CRISPR/Cas9-mediated gene editing in generating patient-specific iPSC models for the study of rare metabolic disorders and their associated complications.

## supplementary material

Below is the link to the electronic supplementary material.

Supplementary Material 1

Supplementary Material 2

Supplementary Material 3

Supplementary Material 4

Supplementary Material 5

Supplementary Material 6

Supplementary Material 7

## Data Availability

The datasets used and/or analyzed during the current study can be obtained from the corresponding author upon reasonable request.

## Abbreviation

PA: Propionic acidemia
PCC: Propionyl-CoA carboxylase
iPSC: Induced pluripotent stem cell
iPSC-CM: iPSC-Derived Cardiomyocytes
WT: Wild-type
Mut: Mutant
PCCA: Propionyl-CoA carboxylase alpha subunit
PCR: Polymerase chain reaction
RT-PCR: Real-time polymerase chain reaction
ER: Endoplasmic reticulum
EB: Embryoid body
LC-MS/MS: Liquid chromatography-tandem mass spectrometry
