# Supplementary material for: Generation of an isogenic human induced pluripotent stem cell line with a mutant propionyl-CoA carboxylase α subunit
Source: Orphanet J Rare Dis. 2026 Jan 23;21:61. doi: 10.1186/s13023-026-04197-6 (PMC12911109; doi:10.1186/s13023-026-04197-6)
Supplement: Supplementary file 1 — Supplementary Material 1 [file 13023_2026_4197_MOESM1_ESM.pptx]

## Slide 1
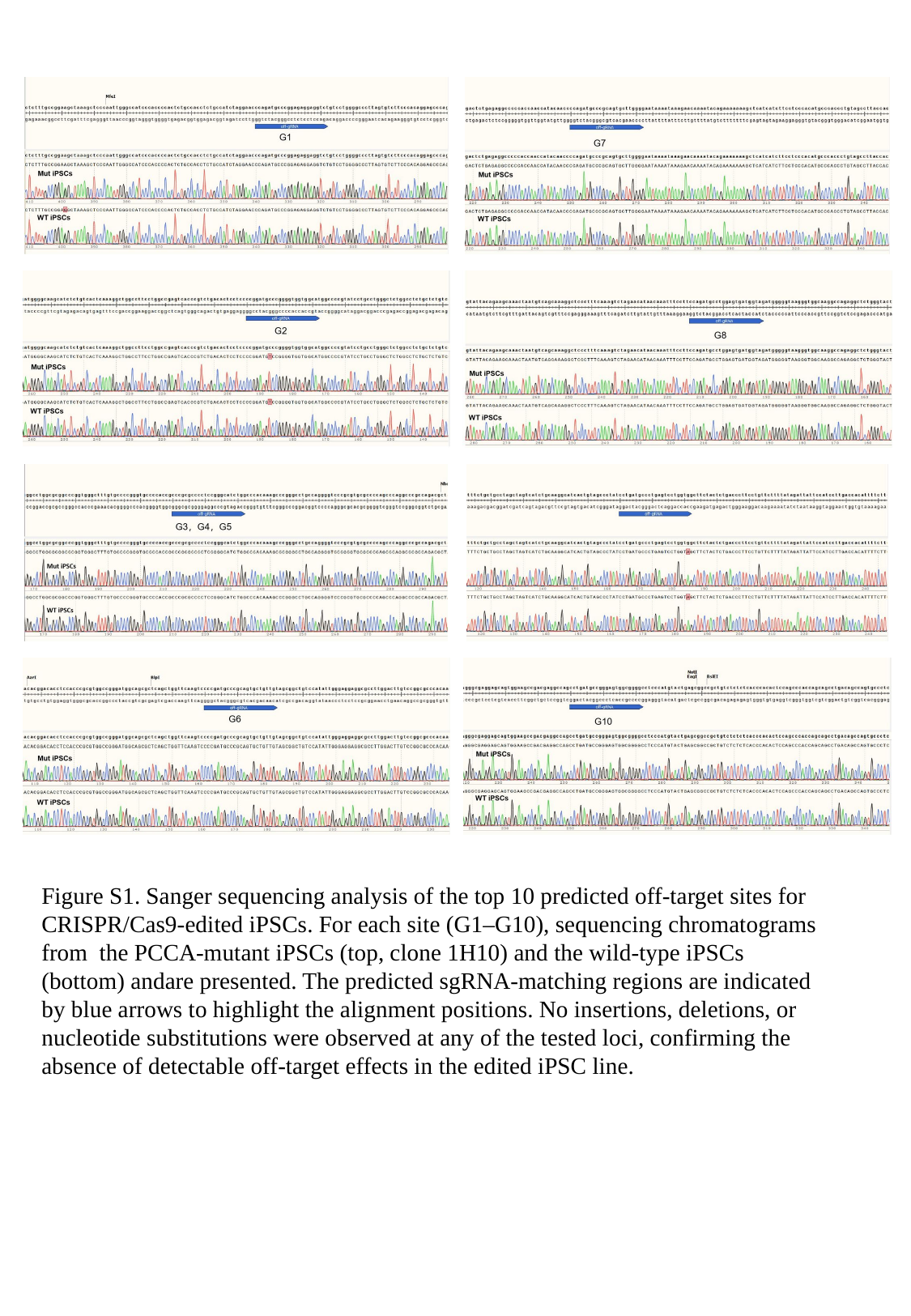

Figure S1. Sanger sequencing analysis of the top 10 predicted off-target sites for CRISPR/Cas9-edited iPSCs. For each site (G1–G10), sequencing chromatograms from the PCCA-mutant iPSCs (top, clone 1H10) and the wild-type iPSCs (bottom) andare presented. The predicted sgRNA-matching regions are indicated by blue arrows to highlight the alignment positions. No insertions, deletions, or nucleotide substitutions were observed at any of the tested loci, confirming the absence of detectable off-target effects in the edited iPSC line.
