# Supplementary material for: Generation of an isogenic human induced pluripotent stem cell line with a mutant propionyl-CoA carboxylase α subunit
Source: Orphanet J Rare Dis. 2026 Jan 23;21:61. doi: 10.1186/s13023-026-04197-6 (PMC12911109; doi:10.1186/s13023-026-04197-6)
Supplement: Supplementary file 2 — Supplementary Material 2 [file 13023_2026_4197_MOESM2_ESM.docx]

| **Off-Target Site** | **Target gRNA Sequence** | **Off-Target Sequence** | **Forward Primer** | **Reverse Primer** | **Amplicon Size (bp)** | **Sequencing Primer** |
| --- | --- | --- | --- | --- | --- | --- |
| G1 | CCACCACCACTCCGGGCATC-GGG | AGACCTCCTCTCCGGGCATCTGG | SMU183-G1-F1, GAACTGTGCTCCTTTTATCTCTGC | SMU183-G1-R1, CTGGTGTAGAAGAAGGAACCTGG | 937 | SMU183-A1-1-R1 |
| G2 | CCACCACCACTCCGGGCATC-GGG | ATGCCACCACCCCGGGCATCCGG | SMU183-G2-F1, TGGATCTGCACGTTATACAGAAAGC | SMU183-G2-R1, ATCATCCAGAAAGAGGTGTGGAGA | 973 | SMU183-A1-2-R1 |
| G3 | CCACCACCACTCCGGGCATC-GGG | CCCGCGCCCCTCCGGGCATCTGG | SMU183-G3-F1, CTGGAAATCGCAAAATCCTCTAGC | SMU183-G3-R1, GACGTCACAAGCTTCCAAGATG | 655 | SMU183-A1-3-F1 |
| G4 | CCACCACCACTCCGGGCATC-GGG | CCCGCGCCCCTCCGGGCATCTGG | SMU183-G3-F1, CTGGAAATCGCAAAATCCTCTAGC | SMU183-G3-R1, GACGTCACAAGCTTCCAAGATG | 655 | SMU183-A1-4-F1 |
| G5 | CCACCACCACTCCGGGCATC-GGG | CCCGCGCCCCTCCGGGCATCTGG | SMU183-G3-F1, CTGGAAATCGCAAAATCCTCTAGC | SMU183-G3-R1, GACGTCACAAGCTTCCAAGATG | 655 | SMU183-A1-5-F1 |
| G6 | CCACCACCACTCCGGGCATC-GGG | ACAACAGCACTGCGGGCATCGGG | SMU183-G6-F1, ACTGAGATATTTCCCACTCATGGC | SMU183-G6-R1, GACAAAACCTAAAAGCACGCAGAG | 657 | SMU183-A1-6-F1 |
| G7 | CCACCACCACTCCGGGCATC-GGG | CCCCAAGCACTGCGGGCATCTGG | SMU183-A1-7-F1, CTCAAGCCCTAGTAGCTGAATTTG | SMU183-G7-R1, TGCCTGAAGGATTCTTGGTGATTT | 491 | SMU183-A1-7-F1 |
| G8 | CCACCACCACTCCGGGCATC-GGG | CTACCATCACTCCAGGCATCTGG | SMU183-A1-8-F1, GCAATGAGATCTCTACCTGCCA | SMU183-G8-R1, ACCCTTCATCACAGCTTCATCTT | 680 | SMU183-A1-8-R1 |
| G9 | CCACCACCACTCCGGGCATC-GGG | CCACCAGGACTCAGGGCATCAGG | SMU183-G9-F1, CTTAATTCCTGGCAAGTGGATTGT | SMU183-A1-9-R1, CCTTCAGCCTTGGCTAAGTAAATC | 419 | SMU183-A1-9-F1 |
| G10 | CCACCACCACTCCGGGCATC-GGG | GCCCCGCCACTCCCGGCATCAGG | SMU183-G10-F1, CAAGATGCAATGATGCAAGGACTA | SMU183-A1-10-R1, GTTTTCTTGTCCCTTGTTGGCTTG | 571 | SMU183-A1-10-F1 |

Supplementary Table S1. Primers used for PCR amplification and sequencing of predicted off-target sites
