# Supplementary material for: Generation of an isogenic human induced pluripotent stem cell line with a mutant propionyl-CoA carboxylase α subunit
Source: Orphanet J Rare Dis. 2026 Jan 23;21:61. doi: 10.1186/s13023-026-04197-6 (PMC12911109; doi:10.1186/s13023-026-04197-6)
Supplement: Supplementary file 7 — Supplementary Material 7 [file 13023_2026_4197_MOESM7_ESM.ppt]

## Slide 1
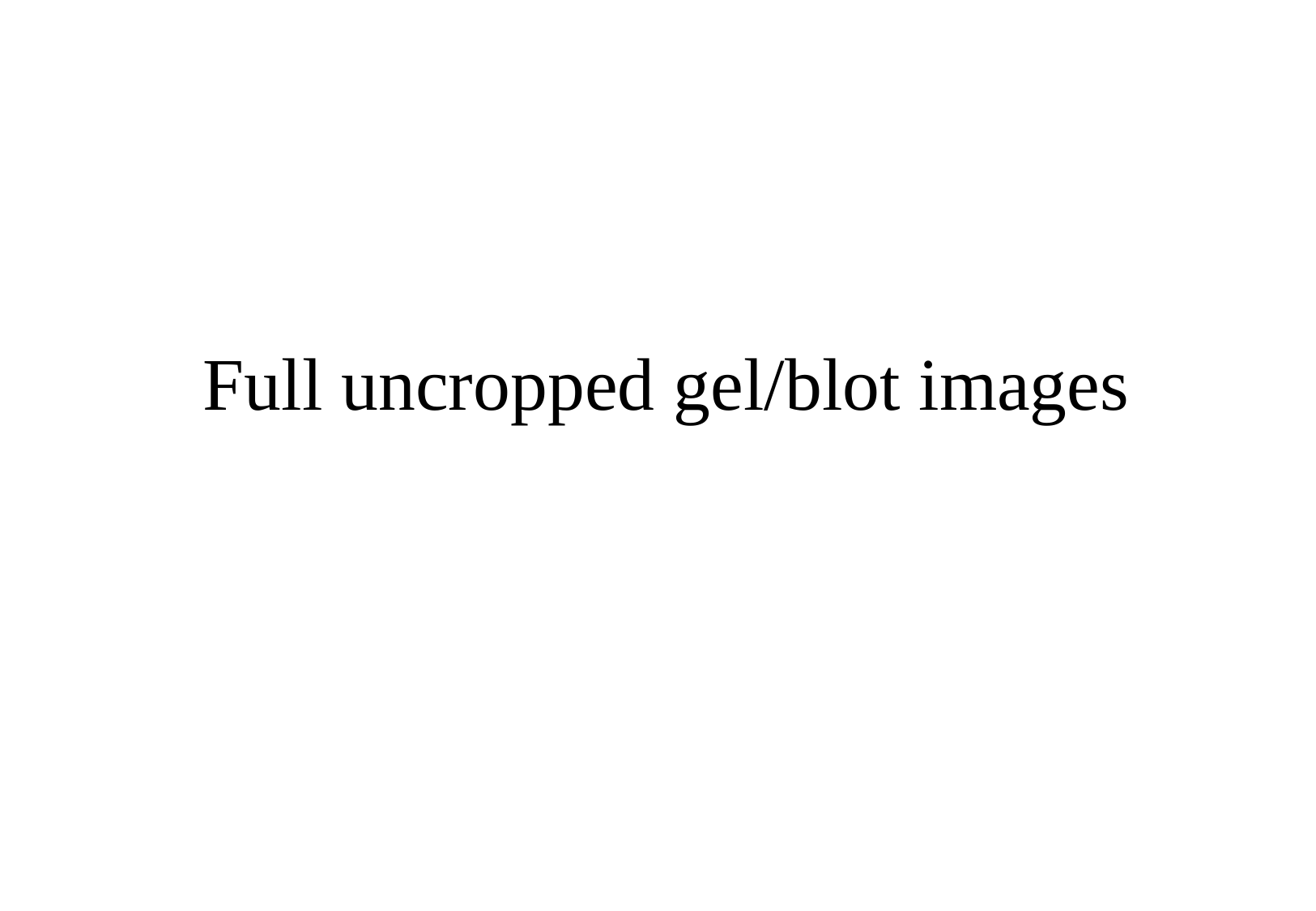

# Full uncropped gel/blot images

## Slide 2
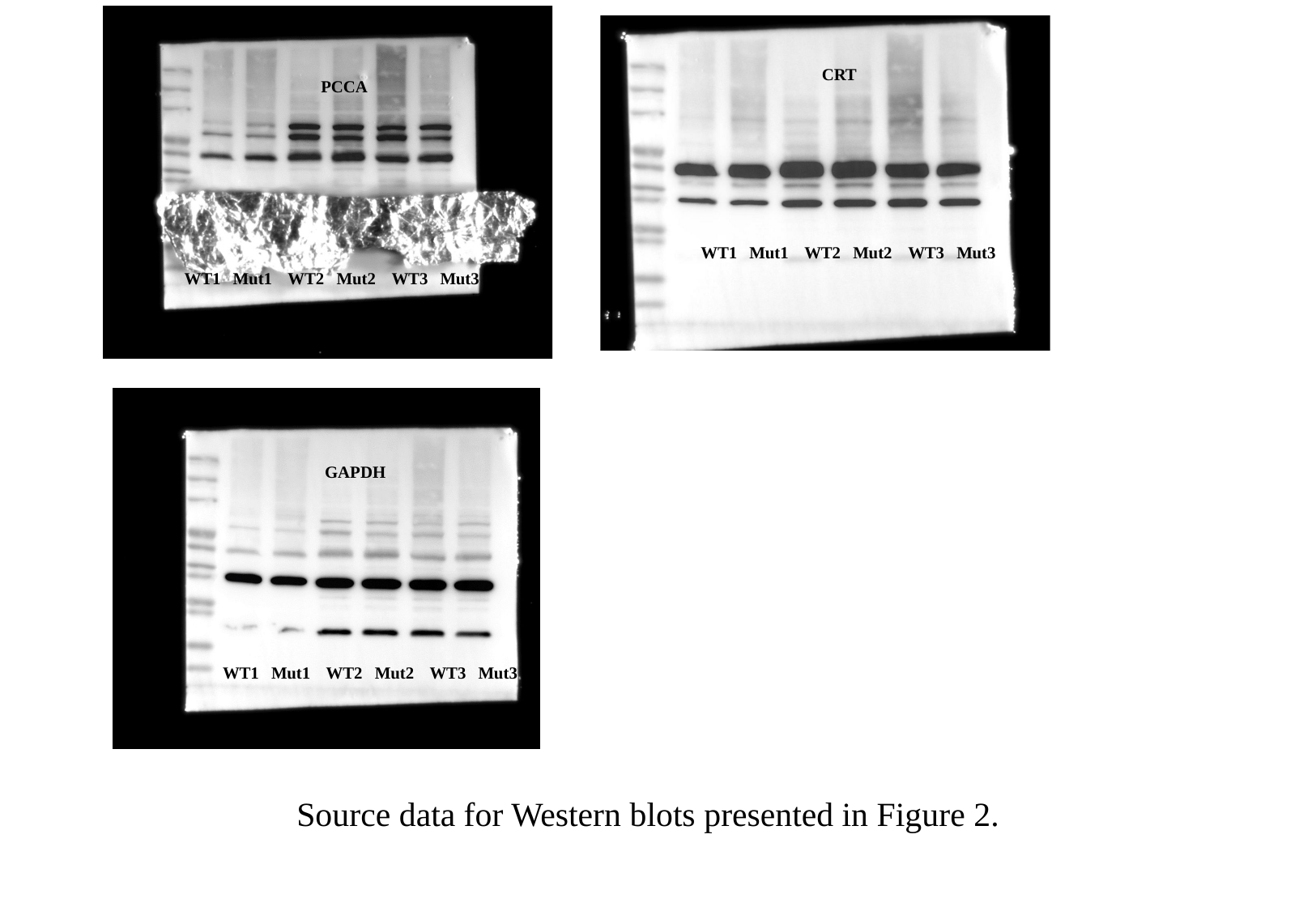

PCCA
CRT
WT1
Mut1
WT2
Mut2
WT3
Mut3
WT1
Mut1
WT2
Mut2
WT3
Mut3
GAPDH
WT1
Mut1
WT2
Mut2
WT3
Mut3
Source data for Western blots presented in Figure 2.

## Slide 3
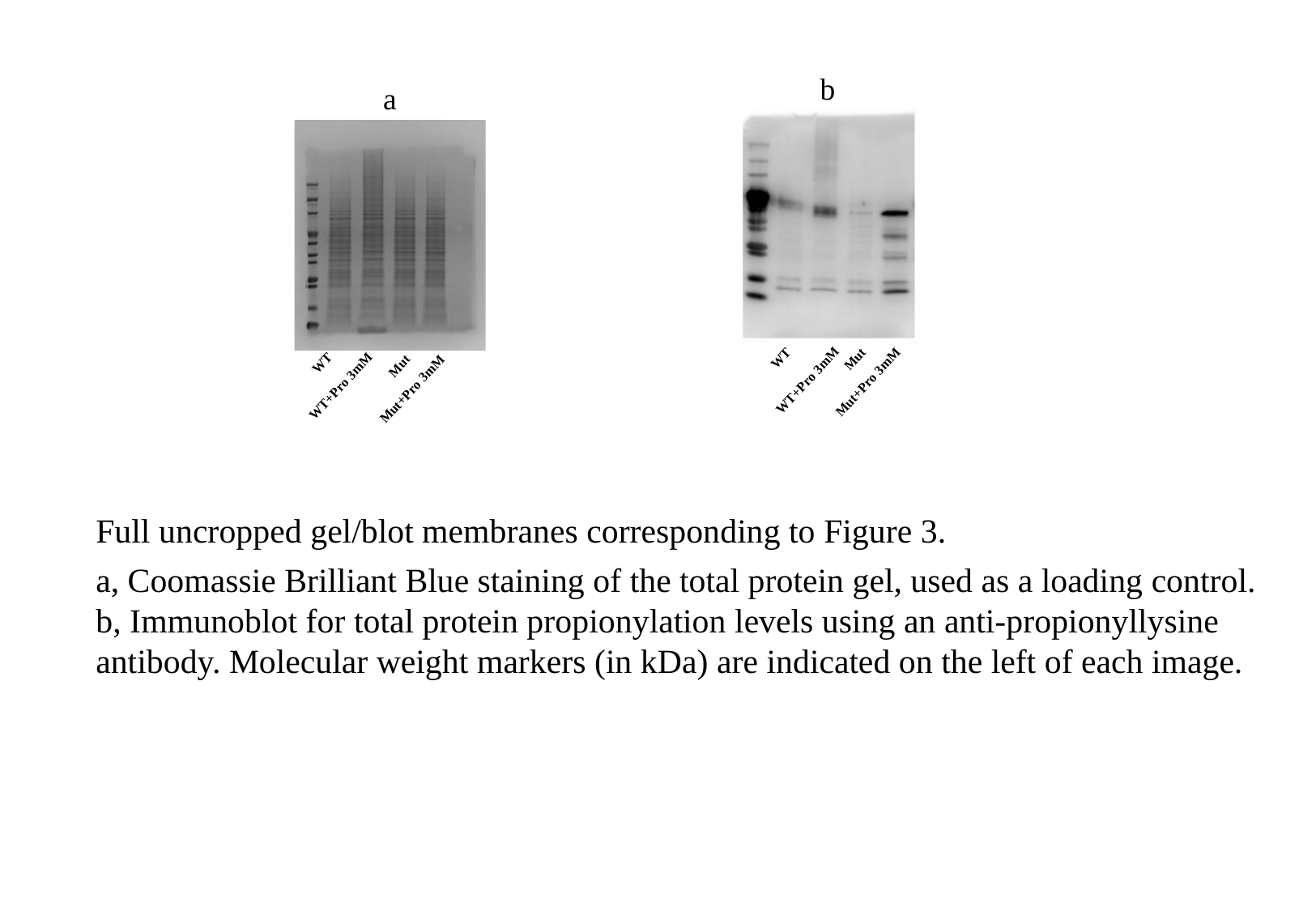

b
a
Mut
WT
WT+Pro 3mM
Mut+Pro 3mM
WT
Mut
WT+Pro 3mM
Mut+Pro 3mM
Full uncropped gel/blot membranes corresponding to Figure 3.
a, Coomassie Brilliant Blue staining of the total protein gel, used as a loading control. b, Immunoblot for total protein propionylation levels using an anti-propionyllysine antibody. Molecular weight markers (in kDa) are indicated on the left of each image.
